# Controlled trial of twelve versus six courses of chemotherapy in the treatment of small-cell lung cancer. Report to the Medical Research Council by its Lung Cancer Working Party.

**DOI:** 10.1038/bjc.1989.118

**Published:** 1989-04

**Authors:** 

## Abstract

A total of 497 patients with histologically or cytologically confirmed small-cell lung cancer were prescribed initial treatment with six courses of etoposide, cyclophosphamide, methotrexate and vincristine at 3-week intervals. Patients with limited disease (74% of the total) also received radiotherapy (40 Gy in 15 fractions in 3 weeks) to the primary site between courses 2 and 3. At the end of this initial treatment, 265 patients still in complete or partial response were randomly allocated to six further courses of maintenance chemotherapy (M series: 131 patients) or to no maintenance chemotherapy (NoM series: 134 patients). Response, as assessed 3 weeks after the second course of initial chemotherapy, was achieved in 85% of the 264 patients assessed, a complete response in 11%. The median survival period from the date of start of chemotherapy was 39 weeks; 154 (31%) of the patients were alive at 1 year, 29 (6%) at 2 years and 17 (3%) at 3 years. The patients' general condition and extent of disease pretreatment correlated significantly with survival. Among the 131 M and 134 NoM patients there was no overall survival advantage to either series (P = 0.27, log rank test), although in 99 patients who had a complete response to initial chemotherapy as assessed at the time of randomisation there was a suggestion that survival was longer in the M series (P less than 0.05, log rank test), the median survival periods from the date of randomisation being 42 weeks for the M and 30 weeks for the NoM patients. Maintenance chemotherapy was associated with additional toxicity and a poorer quality of life as assessed intermittently by clinicians and daily by patients. In conclusion, no worthwhile clinical advantage was achieved by the policy of continuing chemotherapy beyond six courses, except possibly in patients with a complete response to the initial six courses.


Br. J. Cancer (1989), 59, 584–590 © The Macmillan Press Ltd., 1989

### Controlled trial of twelve versus six courses of chemotherapy in the treatment of small-cell lung cancer

### Report to the Medical Research Council by its Lung Cancer Working Party

*Prepared on behalf of the participating members by Professor N.M. Bleehen (Chairman), Mr P.M. Fayers (Statistician), Dr D.J. Girling (Secretary and Coordinator) and Mr R.J. Stephens (Data Manager).*

**Summary** A total of 497 patients with histologically or cytologically confirmed small-cell lung cancer were prescribed initial treatment with six courses of etoposide, cyclophosphamide, methotrexate and vincristine at 3-week intervals. Patients with limited disease (74% of the total) also received radiotherapy (40 Gy in 15 fractions in 3 weeks) to the primary site between courses 2 and 3. At the end of this initial treatment, 265 patients still in complete or partial response were randomly allocated to six further courses of maintenance chemotherapy (M series: 131 patients) or to no maintenance chemotherapy (NoM series: 134 patients). Response, as assessed 3 weeks after the second course of initial chemotherapy, was achieved in 85% of the 264 patients assessed, a complete response in 11%. The median survival period from the date of start of chemotherapy was 39 weeks; 154 (31%) of the patients were alive at 1 year, 29 (6%) at 2 years and 17 (3%) at 3 years. The patients' general condition and extent of disease pretreatment correlated significantly with survival. Among the 131 M and 134 NoM patients there was no overall survival advantage to either series ($P = 0.27$, log rank test), although in 99 patients who had a complete response to initial chemotherapy as assessed at the time of randomisation there was a suggestion that survival was longer in the M series ($P < 0.05$, log rank test), the median survival periods from the date of randomisation being 42 weeks for the M and 30 weeks for the NoM patients. Maintenance chemotherapy was associated with additional toxicity and a poorer quality of life as assessed intermittently by clinicians and daily by patients. In conclusion, no worthwhile clinical advantage was achieved by the policy of continuing chemotherapy beyond six courses, except possibly in patients with a complete response to the initial six courses.

Small-cell lung cancer is usually highly sensitive to multi-drug chemotherapy and radiotherapy (reviewed by Aisner *et al.*, 1983; Greco *et al.*, 1985; Livingston, 1986). Because the disease is known to metastasise early, many clinicians have adopted a treatment policy combining these two modalities in the initial treatment of patients, even when disease at presentation is apparently limited to the primary site and regional lymph nodes (MRC Lung Cancer Working Party, 1979). The role of radiotherapy to the tumour and related lymph nodes additional to chemotherapy remains uncertain: some series have shown that radiotherapy reduces the incidence of local recurrence and prolongs survival, but others suggest that chemotherapy alone gives as good results (reviewed by Bleehen, 1986). The aims of treatment are to control the symptoms of disease and to prolong survival as assessed by median survival time or (more importantly) survival for 2 years or longer, or by local and metastatic relapse-free intervals.

The treatment is troublesome to the patient and may be toxic and it is therefore undesirable to continue treatment for longer than is necessary. If the tumour responds, a maximum response, complete or partial (World Health Organization, 1979), is usually achieved after only two or three courses of chemotherapy, but it is not known how much longer treatment should be continued to obtain maximum sustained therapeutic benefit (reviewed by Greco *et al.*, 1985). The present multicentre randomised study was designed to compare 12 against six courses of the same chemotherapy in the treatment of both limited and extensive disease, the patients with limited disease also receiving radiotherapy to the tumour and related lymph nodes after the second course of chemotherapy.

### Methods

### *Eligibility*

Patients aged 75 years or less were eligible if they had previously untreated, histologically or cytologically proved small-cell lung cancer of any extent, normal renal function, and were able to get out and about, even if activity was restricted, and to walk over 100 yards along the flat without dyspnoea (WHO grade 0–2; World Health Organization, 1979), unless poor performance status was due to an unrelated condition or to a cause, such as inappropriate ADH secretion, likely to respond to chemotherapy. Patients were not eligible if they had some other disease contra-indicating chemotherapy or radiotherapy.

### *Histological or cytological diagnosis*

The diagnosis was made by the histopathologist from the referring centre according to the WHO classification (World Health Organization, 1981) on a specimen obtained from bronchial, pleural, lung, mediastinal or lymph node biopsy, bronchial brushings, or sputum cytology. The specimens were later examined by a single reference histopathologist for confirmation of the cell type.

### *Pretreatment investigations*

The pretreatment investigations included a thorough clinical examination, a postero-anterior chest radiograph, measurement of the blood haemoglobin and urea and plasma creatinine concentrations, and total blood white cell and platelet counts. The extent of disease as assessed on clinical and radiographic evidence was recorded as either limited to the soft tissue of one hemithorax, the mediastinum and the ipsilateral and contralateral scalene and lower cervical lymph nodes (limited disease), or more extensive than this (extensive disease).

### *Initial treatment (all patients)*

All patients were prescribed initial treatment with 6 courses of chemotherapy, each course given on three consecutive days at 3-week intervals. Etoposide 120 mg m$^{-2}$ was given by intravenous infusion over 30 min, together with cyclophosphamide 1 g m$^{-2}$, methotrexate 35 mg m$^{-2}$ and vincristine 1.3 mg m$^{-2}$ (maximum dose 2.0 mg) by intravenous injection on day 1. Etoposide 240 mg m$^{-2}$ by mouth or 120 mg m$^{-2}$ intravenously was given on days 2 and 3. Patients with limited disease were also given megavoltage radiotherapy to a midline dose of 40 Gy in 15 daily fractions over 3 weeks

Correspondence: Dr D.J. Girling, MRC Cardiothoracic Epidemiology Group, Brompton Hospital, Fulham Road, London SW3 6HP, UK.
Received 8 July 1988, and in revised form, 3 November 1988.

between the second and third courses of chemotherapy, starting 3 weeks after the second course. It was delivered through planned portals to the primary site and mediastinal lymph nodes, the field extending at least from the suprasternal notch to 3 cm below the carina and encompassing the full width of the mediastinum and lung hila.

*Allocation to maintenance or no maintenance chemotherapy*

Patients in partial or complete response (that is with an estimated decrease in tumour size of 50% or more (World Health Organization, 1979)) at the time of the fifth course of initial chemotherapy were eligible for random allocation either to six more courses of maintenance chemotherapy (M series) or to no maintenance chemotherapy (NoM series), the allocation being stratified for admitting centre, extent of disease pretreatment and degree of response (partial or complete) at the time of randomisation. Maintenance chemotherapy consisted of the same chemotherapy in the same dosages as before, but starting 4 weeks after the sixth course of initial chemotherapy and at intervals of 4 instead of 3 weeks.

*Reports and investigations*

In addition to a pretreatment report, a report on each patient was completed at each attendance for treatment, then monthly up to 12 months and then once every 3 months. These reports included details of the treatment given, the response to treatment, adverse reactions encountered, metastases, the blood haemoglobin concentration and total white cell and platelet counts. At death, the certified cause was reported and if an autopsy was done, the findings.

*Assessment of quality of life*

At each attendance the clinician recorded his assessment of the patient's overall condition, level of activity and degree of breathlessness according to the categories shown in Table I. In addition, the patients themselves completed a daily diary card (Fayers & Jones, 1983) every evening after their last meal, recording how they had been feeling during the past 24 h, coding their assessments as below.

**Table I** Condition pretreatment of all 497 patients

| Condition | Patients No. | % |
|---|---|---|
| Sex: Male | 326 | 66 |
| Age (years): | | |
| less than 45 | 26 | 5 |
| 45–54 | 94 | 19 |
| 55–64 | 237 | 48 |
| 65 or more | 140 | 28 |
| Extent of disease: Limited | 369 | 74 |
| Overall condition: | | |
| 1. Excellent | 55 | 11 |
| 2. Good | 243 | 50 |
| 3. Fair | 163 | 34 |
| 4. Poor | 23 | 5 |
| 5. Very poor | 2 | <1 |
| Not recorded | 11 | |
| Level of physical activity: | | |
| 1. At work or active retirement | 115 | 24 |
| 2. Full activity but not at work | 154 | 32 |
| 3. Out and about, but activity restricted | 207 | 43 |
| 4. Confined to home or hospital | 4 | 1 |
| 5. Confined to bed | 0 | 0 |
| Not recorded | 17 | |
| Degree of breathlessness: | | |
| 1. Climbs hills or stairs without dyspnoea | 100 | 21 |
| 2. Walks any distance on flat without dyspnoea | 162 | 34 |
| 3. Walks over 100 yards without dyspnoea | 193 | 41 |
| 4. Dyspnoea on walking 100 yards or less | 12 | 3 |
| 5. Dyspnoea on mild exertion, e.g. undressing | 3 | 1 |
| Not recorded | 27 | |

*Vomiting* 1, none; 2, poor appetite; 3, felt sick but wasn't; 4, sick once; 5, sick more than once.

*Activity* 1, normal work/housework; 2, normal work but with effort; 3, reduced activity but not confined to home; 4, confined to home or hospital; 5, confined to bed.

*Mood* 1, very happy; 2, happy; 3, average; 4, miserable; 5, very miserable.

*Anxiety* 1, very calm; 2, calm; 3, average; 4, anxious; 5, very anxious.

*Overall condition* 1, very well; 2, well; 3, fair; 4, poor; 5, very ill.

### Results

*Patients in the study*

Between June 1981 and February 1985, 542 patients were admitted from 26 centres in the United Kingdom; seven patients were excluded because they were not eligible and had been admitted in error and 38 because the reference histopathologist considered that their histology was not small-cell lung cancer. There remain 497 patients for analysis.

Before treatment (Table I) 74% of the patients had limited disease, the overall condition as assessed by the clinicians was excellent or good in 61%, the level of physical activity was normal in 56% and 56% were able to walk any distance along the flat without dyspnoea.

*Initial response and randomisation*

The initial response to treatment was assessed from clinical and radiographic findings alone 3 weeks after the second course of chemotherapy, that is 6 weeks after the start of chemotherapy and before any radiotherapy was given. Of 398 patients alive at that time, 264 were assessed. Of the 264, 225 (85%) had a response, 29 (11%) a complete response.

In all, 295 of the 497 patients completed the initial period of treatment and were still responding, and a further 29 completed but were no longer responding. The remaining 173 did not complete the initial period, the reason being death in 128, inadequate response in 16, refusal in 16 and toxicity in 13. Of the 295, 265 (53% of the 497) were allocated at random to either maintenance (131 patients) or no maintenance (134 patients) chemotherapy. The remaining 30 patients, all potentially eligible for randomisation, were not randomised, the reason being toxicity in 13, refusal in six and continuation of chemotherapy beyond six courses (without randomisation) in three; for the remaining eight no reason was given. Of the 265 patients allocated, 196 (74%) had had limited disease on admission and 99 (37%) had achieved a complete response as assessed at the time of randomisation.

*Chemotherapy received*

During the initial period of treatment (Table II), 278 (56%) of the 497 patients received their chemotherapy without modification. A further 156 (31%) had their chemotherapy modified at the decision of the clinician because of toxicity; 59 (12%) had all their chemotherapy stopped, and the remaining four (1%) never started chemotherapy: three died and the fourth declined all treatment.

Of the 131 patients allocated to receive maintenance chemotherapy, 46 (35%) received it without modification. A further 21 (16%) had their maintenance chemotherapy modified at the decision of the clinician because of toxicity, 31 (24%) had all their maintenance chemotherapy stopped and the remaining 33 (25%) never started maintenance chemotherapy: 18 decided not to continue, 12 began to relapse

**Table II** Chemotherapy received

| Chemotherapy | Initial chemotherapy No. | Initial chemotherapy % | Maintenance chemotherapy No. | Maintenance chemotherapy % |
|---|---|---|---|---|
| Received without modification | | | | |
| All six courses | 157 | | 22 | |
| Until no longer responding | 30 | | 16 | |
| Until death | 91 | | 8 | |
| Total | 278 | 56 | 46 | 35 |
| Modified because of toxicity | | | | |
| One or more courses delayed | 113 | | 13 | |
| One or more drugs withdrawn | 24 | | 6 | |
| Dosages reduced | 65 | | 5 | |
| Total | 156 | 31 | 21 | 16 |
| All drugs stopped | | | | |
| Toxicity | 31 | | 3 | |
| Patient declined to continue | 23 | | 22 | |
| Clinician decided not to continue | 5 | | 6 | |
| Total | 59 | 12 | 31 | 24 |
| Never started | 4 | 1 | 33 | 25 |
| Total patients | 497 | 100 | 131 | 100 |

shortly after randomisation (five of them dying) and in three their clinician decided it would be inappropriate.

Of the total 497 patients, 159 received additional treatment for relapse, this being chemotherapy alone in 16, radiotherapy alone in 120 and both in 23 patients.

### *Survival*

Follow-up is complete for all 497 patients up to 3 years from the date of start of chemotherapy (or from the date of entry for the four patients who never started chemotherapy). The survival curves from the date of start are shown in Figure 1 according to the level of physical activity pretreatment (as graded in Table I). Survival was significantly longer for the patients with a better grade of activity ($P < 0.001$ log rank test) and the difference was largely accounted for by early deaths in patients graded 3 or 4. Indeed, six (2%) of the patients graded 1 or 2 died during the first 3 weeks compared with 26 (12%) graded 3 or 4 ($P < 0.001$). The median survival period from the date of start of chemotherapy (Table III) was 39 weeks; 154 (31%) of the patients were alive at 1 year, 29 (6%) at 2 years and 17 (3%) at 3 years. To date, 12 of 452 assessable are alive at 4 years, and six of 287 at 5 years.

There is no statistically significant difference between survival in the M and NoM series during 3 years from randomisation ($P = 0.27$, log rank test) (Figure 2), the median survival periods from the date of randomisation being 35 weeks (M) and 29 weeks (NoM). Because 25% of the M patients received no maintenance chemotherapy, the small separation between the survival curves could have represented an underestimate of its true effect. Survival was therefore studied in the 91 M patients alive at 6 months after randomisation (who could have received all six courses). Of the 91, 15 received no maintenance chemotherapy, five received one course, and 10, 12, 8, 12 and 29 received two, three, four, five and six courses, respectively. No evidence of a trend between the duration of survival and the amount of maintenance chemotherapy actually received was found, further evidence that prolonging chemotherapy beyond six courses did not, in general, influence survival.

Separate analyses of four subgroups of the 265 randomised patients were made, namely the 196 with limited, the 69 with extensive disease pretreatment, the 166 with partial and the 99 with complete response to chemotherapy at the time of randomisation. The only subgroup in which there was a suggestion that maintenance chemotherapy influenced survival was patients who had had a complete response, among whom it prolonged survival ($P < 0.05$, log rank test). The median survival periods from the date of randomisation within this subgroup were 42 weeks for the M and 30 weeks for the NoM patients. Thus there may possibly be a small advantage from continuing chemotherapy beyond six courses in patients still showing complete response at the end of their initial six courses of treatment.

**Figure 1** Survival from date of start of chemotherapy according to the level of physical activity of the patients pretreatment.

**Figure 2** Survival from the date of start of chemotherapy and from the date of allocation in the 131 M and 134 NoM patients.

**Table III** Survival from date of start of chemotherapy according to the level of physical activity pretreatment

| Level of physical activity | Patients assessed | Median survival (weeks) | Patients alive at: 1 year No. | 1 year % | 2 years No. | 2 years % | 3 years No. | 3 years % |
|---|---|---|---|---|---|---|---|---|
| 1 or 2 | 269 | 44 | 96 | 36 | 22 | 8 | 14 | 5 |
| 3 or 4 | 211 | 33 | 55 | 26 | 7 | 3 | 3 | 1 |
| Not assessed | 17 | – | 3 | 18 | 0 | 0 | 0 | 0 |
| Total | 497 | 39 (37–43) | 154 | 31 (27–35) | 29 | 6 (4–8) | 17 | 3 (2–5) |

Figures in parentheses are 95% confidence limits.

*Prognostic factors*

A proportional hazards model (Cox, 1972) was used to determine the factors relating to survival for (1) all patients and (2) those surviving long enough to be eligible for the M/NoM randomisation, whether or not they were randomized. The pretreatment factors considered in all patients were sex, age, extent of disease, haemoglobin concentration, white cell and platelet counts, height, weight, surface area, general condition, level of physical activity and degree of breathlessness. The most important factor was general condition ($P<0.001$) but adding extent of disease ($P<0.005$) improved the prognostic discrimination.

The median time of randomisation was 145 days from the date of start of chemotherapy and 369 patients (74% of the original 497) who survived to this time were included in the second analysis. All the above factors were considered, along with initial response to treatment and, at the time of randomisation, weight, haemoglobin concentration, white cell and platelet counts, general condition, level of physical activity and degree of breathlessness, and the main adverse reactions. The most important factor was response to treatment ($P < 0.001$), but adding extent of disease pretreatment ($P < 0.001$) and level of physical activity at time of randomisation ($P < 0.005$), improved the prognostic discrimination.

Even allowing for the above prognostic factors, there was no significant benefit from maintenance chemotherapy.

*Time of death related to chemotherapy*

There were 95 patients who died within 4 weeks after the start of a course of initial or maintenance chemotherapy. Of these, 13 died in the first week after the start of the course, 55 in the second, 14 in the third and 13 in the fourth week ($P < 0.001$, $\chi^2$ test). The 95 included 41 who died within 4 weeks after the start of the first course of initial chemotherapy. Of the 41, nine died in the first, 22 in the second, five in the third and five in the fourth week. There was thus an unexpectedly high risk of death during the second week after the start of a course of chemotherapy.

*Cause of death*

Among the 480 patients who died during the 3 years, carcinoma was the certified underlying cause of death in all except eight, in whom it was myocardial infarction in four, and in the remaining four cerebrovascular accident, ruptured aortic aneurysm, perforated gastric ulcer and bronchopneumonia (in the absence of any evidence of residual cancer). Of the 480 patients, 312 (65%) had evidence of persistence or recurrence of tumour at the primary site. Of these, 228 also had distant metastases. For only 25 of the 312 and 22 of the 228 did this evidence include autopsy findings. In all, 378 (79%) of the 480 patients had evidence of distant metastases at the time of death.

It was thought that treatment may have caused or hastened death in 15 (3% of 497) patients, the initial chemotherapy in 11 and maintenance chemotherapy in four. Seven of the 15 died with septicaemia, four with bronchopneumonia, one with marrow depression and one with radiation pericarditis, and two died suddenly and unexpectedly within 10 days of a course of chemotherapy.

*Metastases in patients with limited disease on admission*

Table IV presents an analysis of the first appearance of distant metastases from the time of randomisation to maintenance or no maintenance chemotherapy. There was a suggestion that brain metastases may have been slightly delayed in the M series but this is not statistically significant ($P = 0.22$, log rank test). The findings for the other sites and for metastases at any site were very similar for the two series. These observations provide further evidence that prolonging chemotherapy beyond six courses brought no worthwhile therapeutic benefit.

**Table IV** Occurrence of distant metastases from date of randomisation in 78 M and 82 NoM patients with no evidence of distant metastases at time of randomisation

| Site of metastases | *Cumulative percentage of patients with distant metastases at the following times from randomisation* 6 months | | 12 months | | 24 months | |
|---|---|---|---|---|---|---|
| | *M* | *NoM* | *M* | *NoM* | *M* | *NoM* |
| Liver | 15 | 12 | 27 | 26 | 33 | 29 |
| Brain | 9 | 23 | 27 | 35 | 32 | 39 |
| Bone | 18 | 16 | 26 | 27 | 28 | 29 |
| Other | 8 | 10 | 13 | 12 | 14 | 15 |
| Any site | 36 | 40 | 64 | 62 | 72 | 68 |

**Table V** Main adverse reactions other than alopecia reported during the initial period of chemotherapy

| *Reaction* | *Patients No.* | *%* |
|---|---|---|
| Nausea without vomiting | 71 | 14 |
| Vomiting | 233 | 47 |
| Diarrhoea | 43 | 9 |
| Dysphagia | 16 | 3 |
| Mouth ulcers | 55 | 11 |
| Rash | 30 | 6 |
| Paraesthesia | 53 | 11 |
| Peripheral neuropathy | 28 | 6 |
| Cystitis | 4 | 1 |
| Haematological (WHO grade 2 or worse)[a] | | |
| Total | 176 (223) | 35 (45) |
| Anaemia (Hb 9.4 $g dl^{-1}$ or less) | 105 (118) | 21 (24) |
| Leucopenia (WBC $2.9 \times 10^3 mm^{-3}$ or less) | 100 (148) | 20 (30) |
| Thrombocytopenia (platelets $74 \times 10^3 mm^{-3}$ or less) | 20 (43) | 4 (9) |
| Total patients with reactions | 410 | 82 |
| Total patients | 497 | 100 |

[a]Patients with haematological toxicity at routine assessments are shown. The figures in parentheses include patients with toxicity at all assessments, whether routine or not.

*Adverse reactions*

The main adverse reactions, other than alopecia, that were reported during the initial period of chemotherapy are shown in Table V. Haematological toxicity of WHO grade 2 (World Health Organization, 1979) or worse was reported at routine assessments immediately before a course of chemotherapy in 35% of patients. Additional blood counts were done, however, if there was concern about a patient's progress. When these additional results are included, haematological toxicity was reported in 45% of the patients. In addition, 17 (3%) patients had episodes of septicaemia attributed to drug-induced leucopenia and 98 (20%) were given blood transfusions for anaemia.

During the period after randomisation, adverse reactions were reported in 80 (61%) of the 131 M compared with 16 (12%) of the 134 NoM patients ($P < 0.0001$). In the M series, nausea without vomiting was reported in 45 (34%),

**Table VI** Effect of radiotherapy on platelet counts in 107 patients who received radiotherapy (RT) and 73 who did not (No RT), limited to patients who had platelet counts available at all five assessments

| *Assessment* RT | *No RT* | *Mean platelet count ($1000 mm^{-3}$) $\pm 1.96$ standard error* RT | *No RT* |
|---|---|---|---|
| Before course 1 | Before course 1 | 328 (304–352) | 369 (332–407) |
| Before course 2 | Before course 2 | 420 (387–453) | 454 (401–506) |
| At start of RT | Before course 3 | 414 (380–448) | 452 (407–498) |
| At end of RT | Before course 4 | 215 (119–230) | 438 (396–480) |
| Before course 3 | Before course 5 | 262 (245–280) | 410 (373–446) |

vomiting in 40 (31%), paraesthesiae in 17 (13%), peripheral neuropathy in 10 (8%) and mouth ulcers in nine (7%). The corresponding results for the NoM series were four (3%), three (2%), none, two (1%) and one (1%). Septicaemia was reported in six of the M compared with one of the NoM patients. Thus, toxicity was considerably reduced by stopping chemotherapy after six courses.

There were 116 patients who received radiotherapy between the second and third courses of chemotherapy and who had their platelet counts measured at all five of the routine assessments before the first, second and third courses of chemotherapy and at the start and end of radiotherapy. Their platelet counts were compared (Table VI) with those of patients who did not receive radiotherapy but who had their platelet counts measured at all five of comparable routine assessments, namely before the first five courses of chemotherapy. The mean platelet counts were considerably reduced at the end of radiotherapy and 3 weeks afterwards but were unchanged at comparable assessments in the patients who did not receive radiotherapy. There were no equivalent differences attributable to radiotherapy in the white cell counts or haemoglobin concentrations (details not shown).

### *Quality of life*

The findings on quality of life are to be reported and discussed in detail elsewhere. The main findings during the M/NoM comparison are summarised here.

*Clinicians' assessments* The overall condition of the patients, their level of physical activity and degree of breathlessness as recorded by the clinicians when the patients allocated to the M/NoM comparison attended for assessment at 3 weeks, 6 weeks, 3 months and 6 months from the date of randomisation are shown in Table VII. The proportions were similar for the two series initially, but by 6 months there was a clear advantage to the NoM patients, higher proportions being assigned the better categories and lower proportions the worse categories for all three assessments, namely overall condition ($P < 0.05$), level of physical activity ($P < 0.01$) and degree of breathlessness ($P < 0.05$).

*Compliance in the use of the daily diary cards* The intention was that diary cards should be completed daily by the patients from admission until approaching death. From the date of randomisation, 94 (35%) of the 265 patients returned no cards at all, 35 (13%) returned cards covering 1–25% of their survival time or of 6 months from randomisation (whichever was shorter), 31 (12%) 26–50% of this period and 105 (40%) 51–100%.

*Quality of life as recorded by patients* The quality of life during the 6-week period starting 3 weeks after the sixth course of chemotherapy as recorded by patients on their daily diary cards is expressed, in Table VIII, in terms of the percentage of patient-days for each category. This analysis is based on the 64 M and 45 NoM patients who provided at

**Table VII** Clinicians' assessments of overall condition, level of activity, and degree of breathlessness from allocation to maintenance or no maintenance chemotherapy

| | *Percentage of patients at the following times from date of allocation* | | | | | | | |
|---|---|---|---|---|---|---|---|---|
| | *3 weeks* | | *6 weeks* | | *3 months* | | *6 months* | |
| *Assessment* | *M* | *NoM* | *M* | *NoM* | *M* | *NoM* | *M* | *NoM* |
| Overall condition: | | | | | | | | |
| 1. Excellent | 15 | 16 | 13 | 21 | 10 | 23 | 14 | 26 |
| 2. Good | 52 | 42 | 53 | 49 | 48 | 47 | 28 | 43 |
| 3. Fair | 28 | 33 | 32 | 19 | 34 | 17 | 41 | 13 |
| 4. Poor | 4 | 8 | 1 | 7 | 9 | 10 | 13 | 13 |
| 5. Very poor | 0 | 1 | 0 | 4 | 0 | 2 | 4 | 4 |
| Level of physical activity: | | | | | | | | |
| 1. At work or active retirement | 18 | 22 | 16 | 25 | 21 | 30 | 18 | 37 |
| 2. Full activity but not at work | 40 | 30 | 40 | 38 | 28 | 23 | 23 | 27 |
| 3. Out and about but activity restricted | 32 | 39 | 39 | 25 | 41 | 33 | 41 | 27 |
| 4. Confined to home or hospital | 10 | 8 | 5 | 6 | 10 | 10 | 14 | 8 |
| 5. Confined to bed | 0 | 1 | 0 | 6 | 0 | 3 | 4 | 2 |
| Degree of breathlessness: | | | | | | | | |
| 1. Climbs hills or stairs without dyspnoea | 35 | 36 | 26 | 33 | 31 | 31 | 23 | 47 |
| 2. Walks any distance on flat without dyspnoea | 22 | 29 | 34 | 30 | 28 | 37 | 28 | 16 |
| 3. Walks over 100 yards without dyspnoea | 29 | 21 | 25 | 17 | 23 | 21 | 28 | 25 |
| 4. Dyspnoea on walking 100 yards or less | 10 | 12 | 14 | 16 | 11 | 8 | 13 | 8 |
| 5. Dyspnoea on mild exertion, e.g. undressing | 3 | 2 | 1 | 4 | 7 | 2 | 9 | 4 |
| Total patients assessed | 92 | 92 | 77 | 72 | 103 | 86 | 71 | 53 |
| Total patients alive | 128 | 133 | 127 | 133 | 119 | 114 | 91 | 73 |

**Table VIII** Patients' assessments of quality of life during the 6-week period starting 3 weeks after the sixth course of chemotherapy, based on the 64 M and 45 NoM patients with at least 50% of relevant data available

| | *Percentage of patient-days* | | | | | | | | | |
|---|---|---|---|---|---|---|---|---|---|---|
| *Category recorded on diary card*[a] | *Vomiting* | | *Activity* | | *Mood* | | *Anxiety* | | *Overall condition* | |
| | *M* | *NoM* | *M* | *NoM* | *M* | *NoM* | *M* | *NoM* | *M* | *NoM* |
| 1 | 78 | 91 | 19 | 30 | 8 | 15 | 6 | 19 | 11 | 20 |
| 2 | 10 | 4 | 19 | 28 | 30 | 35 | 39 | 39 | 33 | 45 |
| 3 | 5 | 3 | 41 | 37 | 51 | 39 | 43 | 33 | 45 | 30 |
| 4 | 3 | 0 | 18 | 5 | 9 | 11 | 10 | 10 | 10 | 6 |
| 5 | 4 | 1 | 3 | 0 | 1 | 0 | 3 | 0 | 1 | 0 |

[a]The categories are listed in the text.

least 50% of the relevant data. The proportion of patient-days for the two best categories combined (categories 1 and 2) was higher for the NoM series for all five factors, and for the two worst categories combined (categories 4 and 5) was higher for the M series for all factors except mood.

Thus, quality of life from the time of the M/NoM randomisation was better for the NoM patients, as assessed both intermittently by the clinicians and daily by the patients, further reason for not continuing chemotherapy beyond six courses.

### Discussion

This study has shown that when the present chemotherapy regimen of etoposide, cyclophosphamide, methotrexate and vincristine is used in the treatment of limited or extensive small-cell lung cancer, no important overall therapeutic advantage is to be gained from a policy of continuing chemotherapy beyond six courses in patients still showing response at that time. Of 497 eligible patients admitted to the study, 265 completed their initial six courses of chemotherapy and were randomised to a further six courses of maintenance chemotherapy (131 patients) or to no further chemotherapy (134 patients) until relapse, the randomisation being stratified for admitting centre, extent of disease pretreatment and degree of response at the time of randomisation. In a straight comparison between the two groups of patients, there was no evidence of a difference in survival during 3 years of follow-up ($P = 0.27$, log rank test) and control of the primary cancer and of distant metastases was very similar. Of the 131 patients allocated to receive maintenance chemotherapy, only 35% received it without modification. A further 16% had it modified because of toxicity and 24% had it stopped; the remaining 25% never started. Nevertheless, there was no evidence of a trend between the duration of survival and the amount of maintenance chemotherapy actually received.

In analyses of subgroups of patients, there was a suggestion that maintenance chemotherapy may have prolonged survival among 99 patients with a complete response to their initial chemotherapy as assessed at the time of randomisation ($P<0.05$, log rank test). This finding should not, however, be regarded as conclusive, because comparing subgroups increases the likelihood of obtaining such a difference by chance (Simon, 1982). Also, in the 91 patients (complete and partial responders) who were allocated to maintenance chemotherapy and who were still alive 6 months later, there was again no evidence of a trend between duration of survival and amount of maintenance chemotherapy actually received.

During the period of chemotherapy, deaths were not evenly distributed during and between courses. They were most likely to occur during the second week after the date of start of the last course before death, that is, when the white cell count was likely to have been at its lowest. This finding is similar to that observed by Souhami and his colleagues who also found that such deaths were more likely to occur in patients with other unfavourable prognostic factors present pretreatment, especially poor performance status, hepatomegaly, and abnormal liver function tests (Souhami *et al.*, 1988). In the present study, 26 of 32 assessable deaths during the first 3 weeks of the study occurred in patients with a poor performance status on admission (activity grade 3 or 4). Souhami and his colleagues (personal communication) have shown that this high risk of early death in patients with a poor performance status can be greatly reduced by giving antibiotics prophylactically during the early weeks of chemotherapy. In the present study such prophylaxis was left to the individual clinician to decide.

Several drug schedules have been used in attempts to improve the results of therapy for small-cell lung cancer. They include alternating non-cross-resistant combinations, very high dose chemotherapy with autologous marrow rescue, regimens of alternating chemotherapy and radiotherapy, and maintenance chemotherapy after an initial induction course using either the same or different drugs (reviewed by Aisner *et al.*, 1983; Greco *et al.*, 1985; Livingston, 1986). There is little if any evidence for any major improvement in survival as a result of these treatments compared with the more conventional ones, except in some small non-randomised studies.

The present study solely considers the duration of conventional treatment with the same combination chemotherapy throughout. Three other studies have addressed this same question. In a CALGB study of 258 patients (Maurer *et al.*, 1980), it was reported that maintenance chemotherapy significantly prolonged survival in patients with limited disease, with 33% of the patients alive at 24 months compared with 9% who did not receive maintenance chemotherapy. However, this was a complicated study which included three separate randomisations; one limb of the chemotherapy schedule was abandoned during the trial, and patients were randomised to the maintenance and no-maintenance series after achieving a complete response to six courses of induction chemotherapy. Only 46 patients with limited disease achieved a complete response and were eligible for randomisation. The results have also been questioned on the basis of whether the patients in fact received adequate induction therapy (Greco *et al.*, 1985).

In another study of 309 patients conducted by the Midland Small Cell Lung Cancer Group (Cullen *et al.*, 1986), 93 patients who achieved a complete or good response to induction chemotherapy with six courses of vincristine, doxorubicin and cyclophosphamide were randomised to a further eight courses of maintenance chemotherapy or to no further chemotherapy until relapse. Maintenance chemotherapy prolonged survival in the 61 patients with extensive disease on admission ($P = 0.006$, log rank test), the median survival times being 372 compared with 259 days. However, this survival difference was largely, although not entirely, accounted for by differences in performance status and response to induction chemotherapy between the two series. Moreover, in the 32 patients with limited disease, survival was longer in the no maintenance series, although this difference was not statistically significant.

A third study (Harper *et al.*, 1987) reports on a total of 610 evaluable patients with limited or extensive disease initially. They were randomly assigned to eight or four courses of cyclophosphamide, vincristine and etoposide and there was a second randomisation to further chemotherapy with doxorubicin and methotrexate or to symptomatic treatment alone at the time of relapse. There was a small benefit from more prolonged chemotherapy, the median survival times being 39 and 32 weeks ($P = 0.085$), and the relapse-free intervals 31 and 23 weeks ($P < 0.0002$). However, when chemotherapy on relapse was given, the difference was eliminated.

These three studies, together with the one currently reported, do show some evidence of benefit to be gained from prolonging treatment beyond a short induction period in some patients. Other studies using different drugs in the induction and maintenance phases have not shown any appreciable benefit (Woods *et al.*, 1984; Feld *et al.*, 1981, 1984). The question remains as to whether any advantage at all would be seen following more effective short-term regimens initially or on relapse. End-points must not only include short-term survival as expressed by median survival times, but proportions of long-term survivors at 2 and 3 years as in the present study.

The quality of survival is also important. In the present study, potentially troublesome adverse effects were reported in 61% of the patients during maintenance chemotherapy compared with only 12% during the same period in those who were allocated to no further chemotherapy until relapse ($P < 0.0001$), the main types being nausea, vomiting, paraesthesia, peripheral neuropathy and mouth ulcers. Thus, patients can be spared a considerable amount of unpleasant and potentially serious toxicity if the policy is to give no more than six courses of chemotherapy in primary treatment.

The quality of life of the patients was also recorded intermittently by the clinicians and daily by the patients using a diary card during the period of maintenance or no maintenance chemotherapy. According to both the clinicians' assessments, based on overall condition, level of physical activity and degree of breathlessness, and the patients' assessments, based on the severity of nausea and vomiting, level of physical activity, mood, degree of anxiety and overall condition, the quality of life was better for patients allocated not to receive maintenance chemotherapy.

In conclusion, no clear evidence has emerged from this study that survival can be prolonged to a clinically worthwhile extent by a policy of continuing chemotherapy beyond an initial six courses. Also, continuing chemotherapy beyond six courses increases the amount of unpleasant and potentially serious toxicity and adversely affects the quality of patients' lives. In view of the somewhat equivocal findings in the subgroup of patients who had a complete response to their initial chemotherapy, and the findings by Cullen *et al.* (1986) and Harper *et al.* (1987) that maintenance chemotherapy prolonged survival in some groups of patients, further studies on the optimum duration of chemotherapy in the treatment on small-lung cancer are required. The MRC Lung Cancer Working Party is currently comparing six versus three courses of the regimen used in the study reported here.

The following consultants and their colleagues participated in the study: Amersham: A.O. Robson; Bangor: N.G. Hodges; Basingstoke: J.M. Fowler; Belfast: W.P. Abram, J.I. Coyle, W. Craig Martin, J. MacMahon, D.R.T. Shepherd, G. Varghese; Bradford: A.J. King, D.A.G. Newton; Brighton: H.I. Bijapur, J.P.R. Hartley, N. Hodson, C.W. Turton; Bristol: S. Goodman, E.C. Whipp; Cambridge: N.M. Bleehen; Glasgow: G.W. Allan, I. McHattie, A.R. Russell, R.P. Symonds, H.M.A. Yosef; Hammersmith: K.E. Halnan, C.G. McKenzie; Hexham: R.G. Brackenridge, J.B. Ryder; High Wycombe: W.B. Thomson; Ipswich: C.R. Wiltshire; Lanarkshire: J.C.J.L. Bath; Leeds: D.V. Ash, H.J. Close, M.F. Muers, J. Stone; Margate: R.H. Andrews; Merseyside: M.J. Garrett, D.C. Hurman, A.J. Slater; Middlesborough: H.R. Gribbin, N.L.K. Robson, P. Ryan; Middlesex: M. Spittle; Mount Vernon: S. Dische, M.I. Saunders; Newcastle: J.M. Bozzino, G.J. Gibson, D.J. Hendrick, J. Lauckner, S. Nariman; Oxford: M.K. Benson, A.H. Laing, D.J. Lane, R. Marshall; Swindon: J.A. Waddell; Southampton: D.J. Lipscomb, R.D.H. Ryall; Wolverhampton: D.J. Fairlamb; York: A.M. Hunter. Local coordinators were D. Barron, J. Boyle, R. Collins, C. des Rochers, D. Evans, A. Fenwick, S. Jayne, K. McGregor, S. Morrow, S. Mucur, A. Pickett, J. Pye, D. Robinson, G. Sainsbury, M. Stewart, S. Ward and T. Young. The reference histopathologist was Dr P.G.I. Stovin. The trial was coordinated in the Medical Research Council Cardiothoracic Epidemiology Group (until September 1986, Tuberculosis and Chest Diseases Unit) by Dr D.J. Girling assisted by Mr P.M. Fayers and Mr R.J. Stephens. We are grateful to Bristol Myers, Slough, for their assistance with supplies of etoposide.

### References

AISNER, J., ALBERTO, P., BITRAN, J., COMIS, R. & 4 others (1983). Role of chemotherapy in small cell lung cancer: a concensus report of the international association for the study of lung cancer workshop. *Cancer Treat. Rep.*, **67**, 37.

BLEEHEN, N.M. (1986). Radiotherapy for small cell lung cancer. *Chest*, **89**, suppl., 268.

COX, D.R. (1972). Regression models in life tables. *J. R. Stat. Soc., B*, **34**, 187.

CULLEN, M., MORGAN, D., GREGORY, W. & 13 others (1986). Maintenance chemotherapy for anaplastic small cell carcinoma of the bronchus: a randomised, controlled trial. *Cancer Chemother. Pharmacol.*, **17**, 157.

FAYERS, P.M. & JONES, D.R. (1983). Measuring and analysing quality of life in cancer clinical trials: a review. *Stat. Med.*, **2**, 429.

FELD, R., EVANS, W.K., DEBOER, E. & 12 others (1984). Combined modality induction therapy without maintenance chemotherapy for small cell carcinoma of the lung. *J. Clin. Oncol.*, **2**, 294.

FELD, R., PRINGLE, J.F., EVANS, W.K. & 7 others (1981). Combined modality treatment of small cell carcinoma of the lung. *Arch. Int. Med.*, **141**, 469.

GRECO, F.A., JOHNSON, D.H., HAINSWORTH, J.D. & WOLFF, S.N. (1985). Chemotherapy of small-cell lung cancer. *Semin. Oncol.*, **12**, 31.

HARPER, P.G., SOUHAMI, R.L., ASH, C.M., SPIRO, S.G., TOBIAS, J.T. & GEDDES, D. (1987). Treatment duration in small-cell lung cancer: a randomised comparison of 4 versus 8 courses of initial chemotherapy with or without further chemotherapy on relapse. Proceedings of the 4th European Conference on Clinical Oncology and Cancer Nursing, vol. 4. p. 2.

LIVINGSTONE, R.B. (1986). Current chemotherapy of small cell lung cancer. *Chest*, **89**, suppl., 258.

MAURER, L.H., TULLOH, M., WEISS, R.B. & 4 others (1980). A randomised combined modality trial in small cell carcinoma of the lung: comparison of combination chemotherapy-radiation therapy versus cyclophosphamide-radiation therapy, effects of maintenance chemotherapy and prophylactic whole brain irradiation. *Cancer*, **45**, 30.

MEDICAL RESEARCH COUNCIL LUNG CANCER WORKING PARTY (1979). Radiotherapy alone or with chemotherapy in the treatment of small-cell carcinoma off the lung. *Br. J. Cancer*, **40**, 1.

SIMON, R. (1982). Patient subsets and variation in therapeutic efficiency. *Br. J. Clin. Pharmacol.*, **14**, 473.

SOUHAMI, R.L., MORITTU, L., EARL, H.M. & ASH, C.M. (1988). Identification of patients at high risk of chemotherapy-induced toxicity. Proceedings of the IASLC Workshop on Combined Modality Therapy in Lung Cancer, Le Havre, May, 1987.

WOODS, R.L. & LEVI, J.A. (1984). Chemotherapy for small cell lung cancer (SCLC): a randomised study of maintenance therapy with cyclophosphamide, adriamycin, and vincristine (CAV) after remission induction with cisplatinum, VP16-213, and radiotherapy. *Proc. Am. Soc. Clin. Oncol.*, **3**, 214.

WORLD HEALTH ORGANIZATION (1979). *WHO Handbook for Reporting Results of Cancer Treatment.* WHO Offset Publication No. 48. WHO: Geneva.

WORLD HEALTH ORGANIZATION (1981). *International Histological Classification of Tumours No. 1: Histological Typing of Lung Tumours*, second edition. WHO: Geneva.


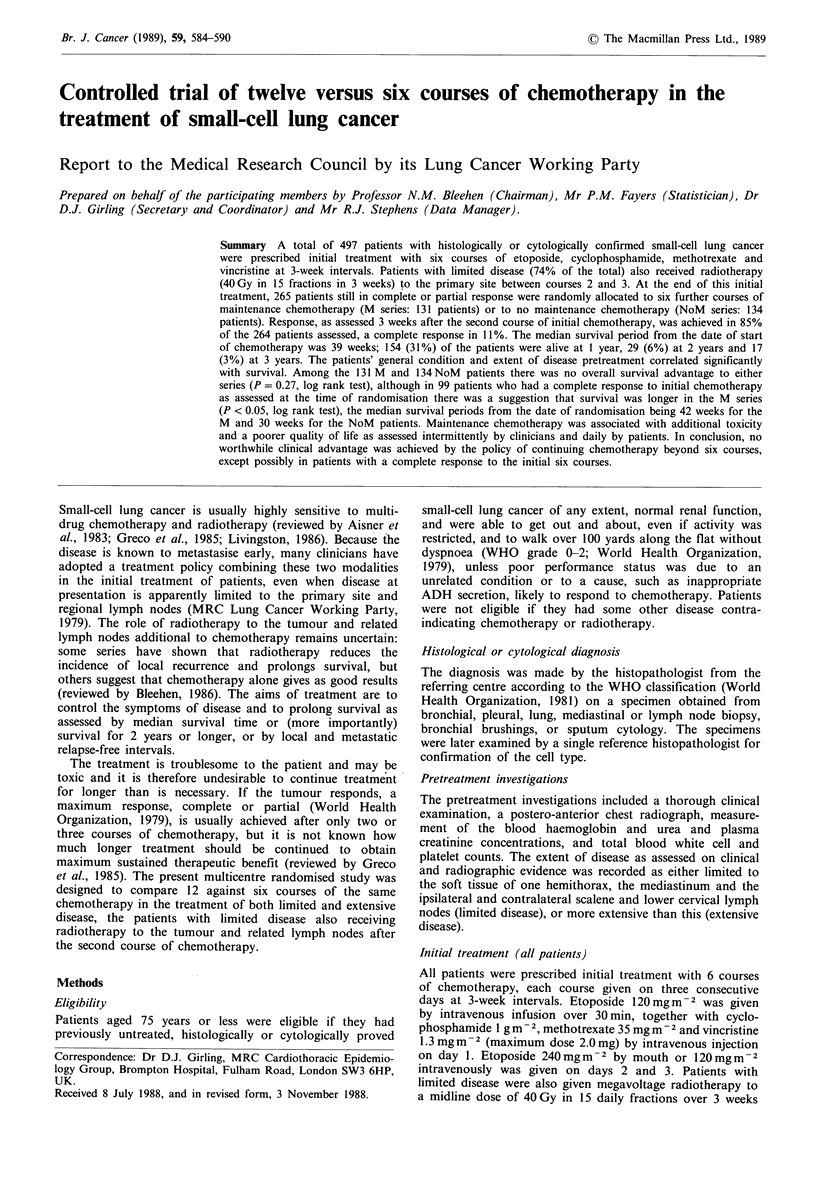

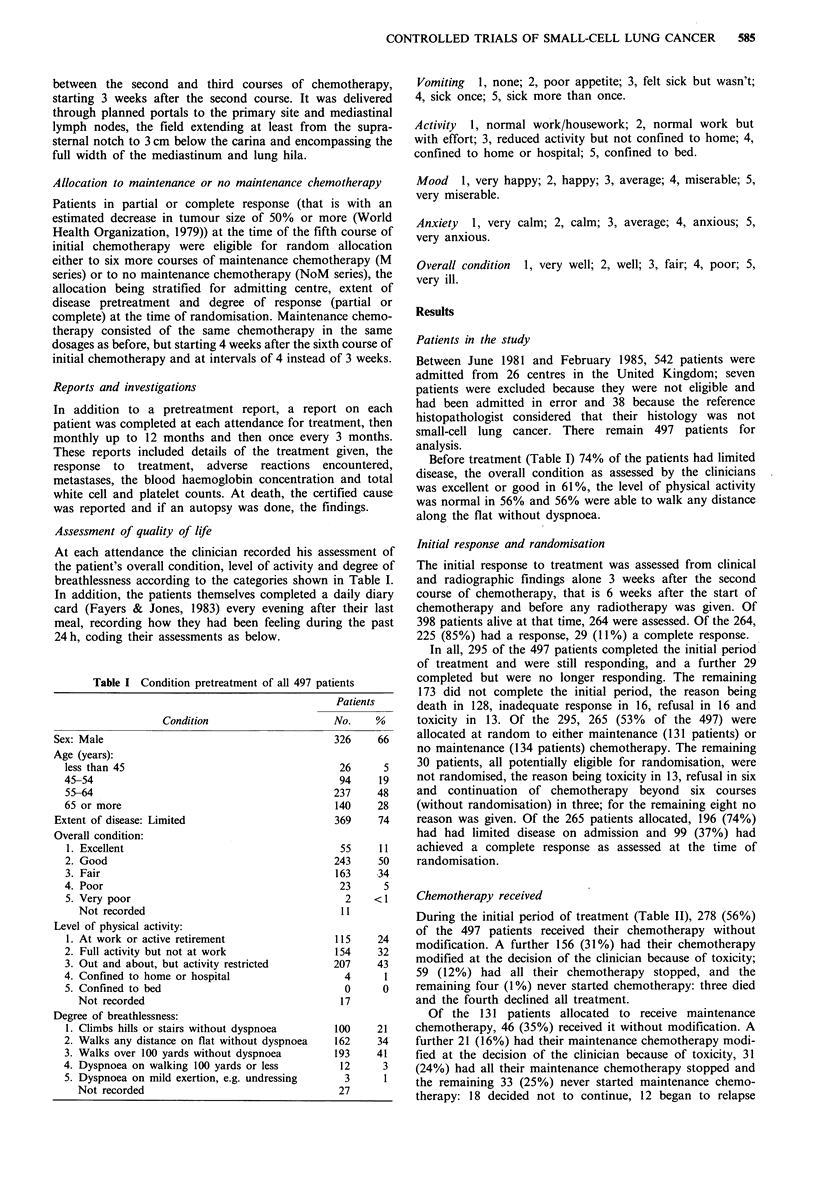

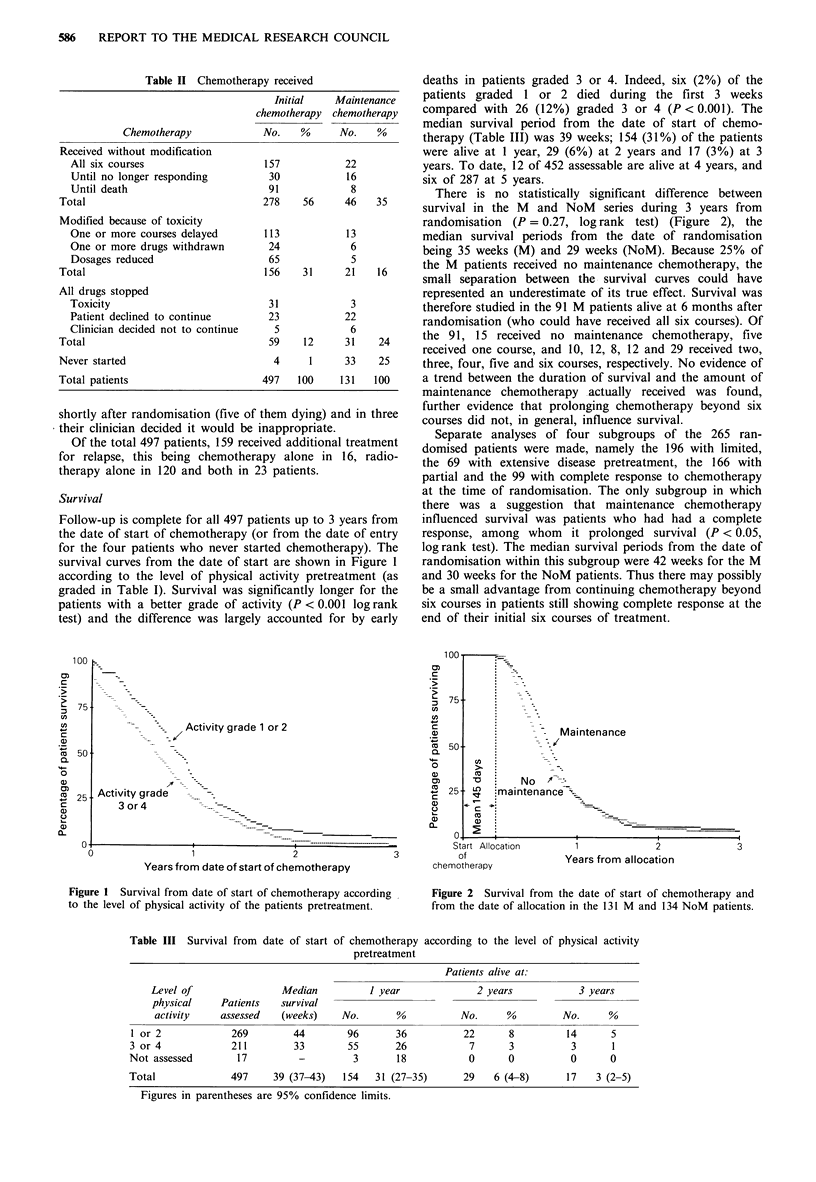

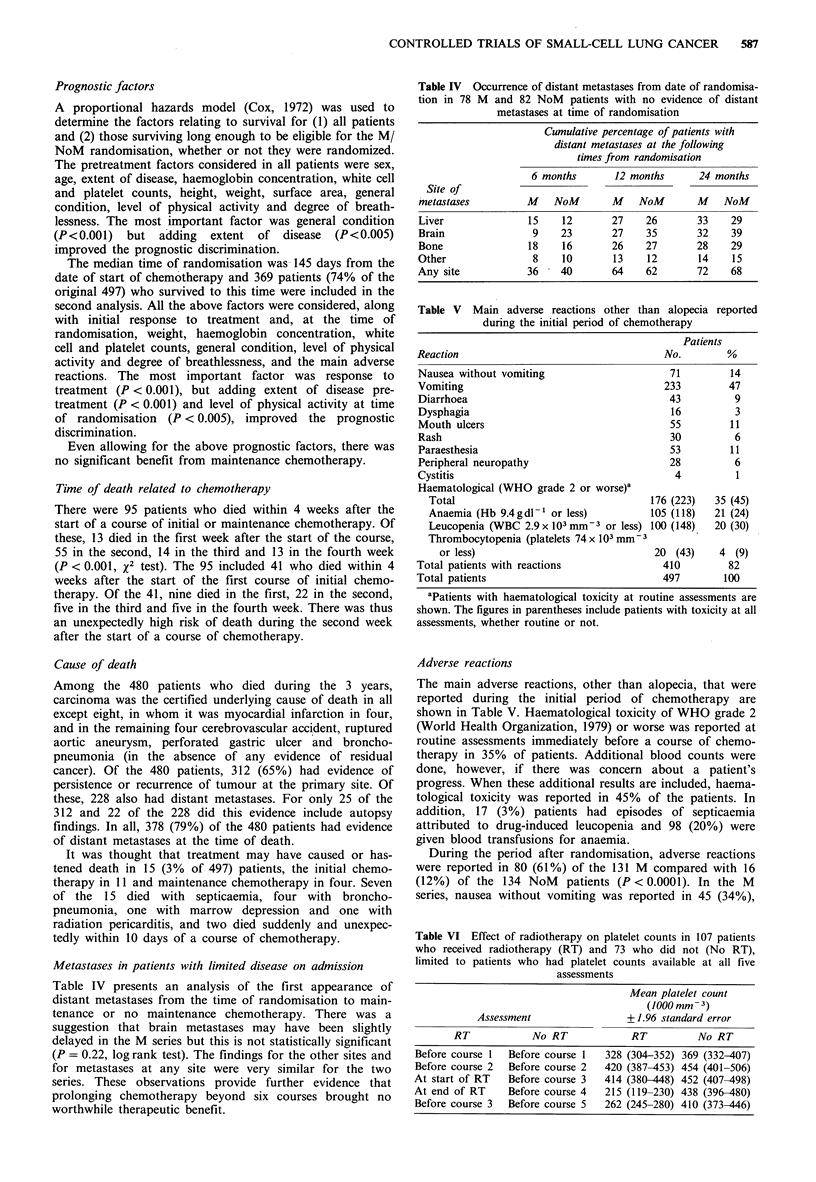

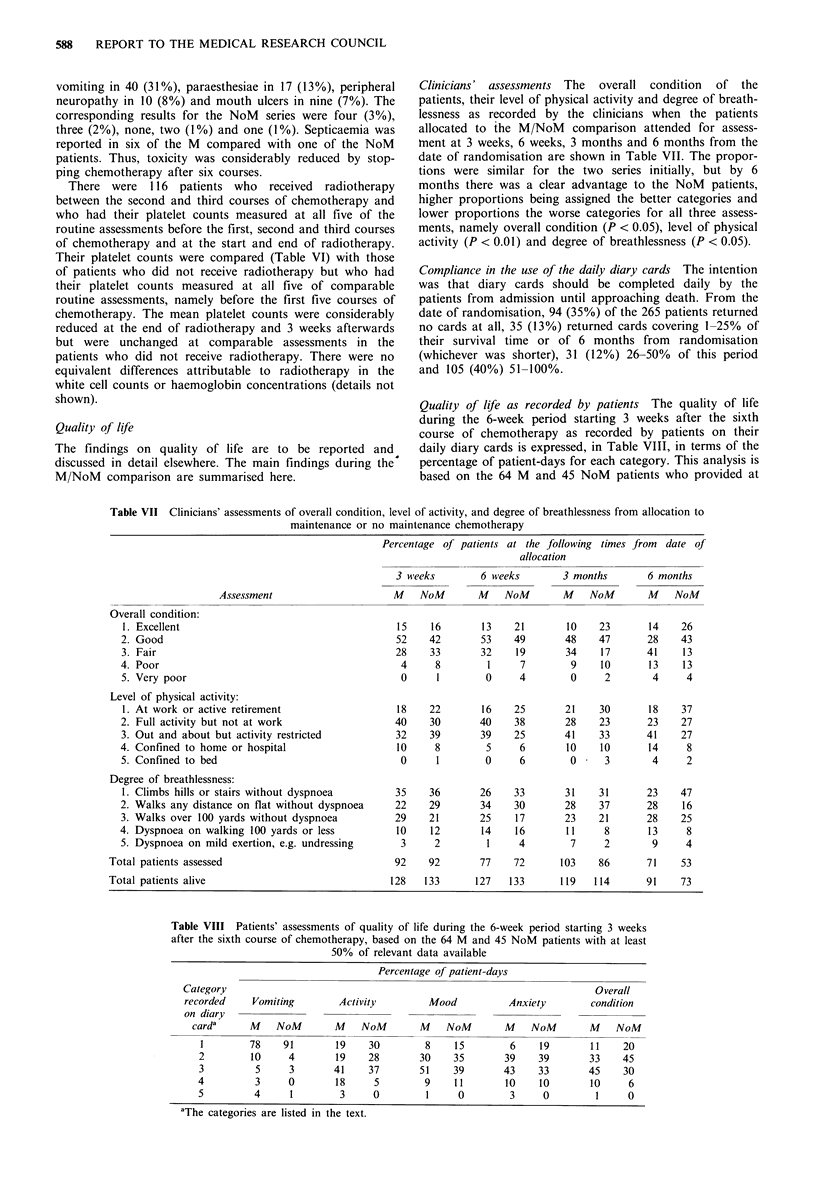

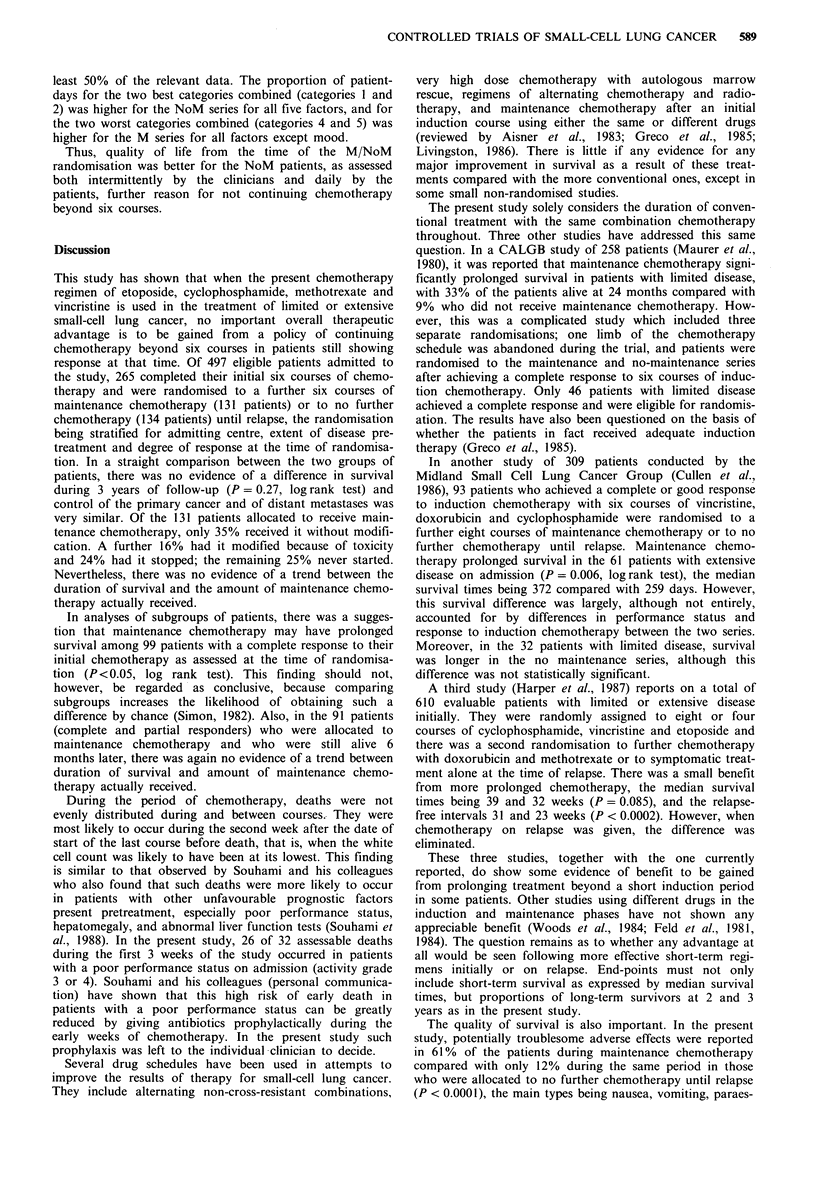

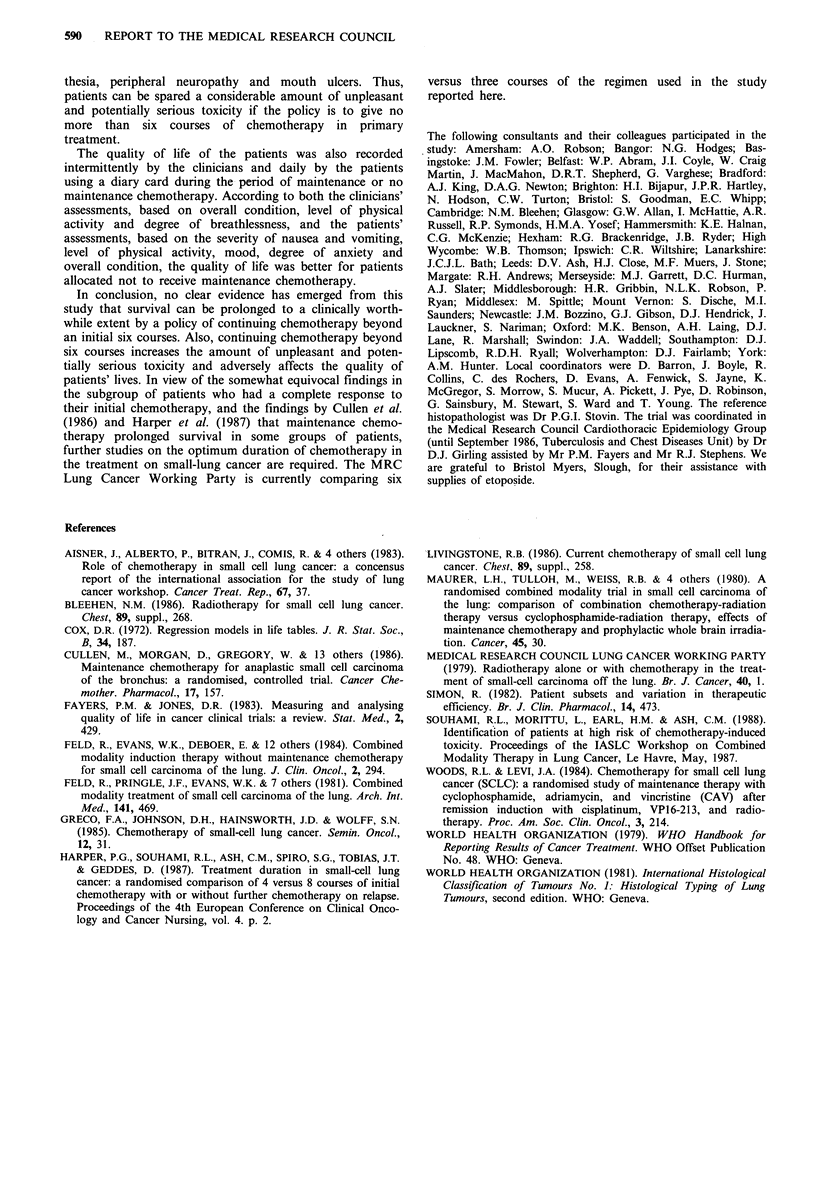


## References

[OCR_01283] Cullen M., Morgan D., Gregory W., Robinson M., Cox D., McGivern D., Ward M., Richards M., Stableforth D., Macfarlane A. (1986). Maintenance chemotherapy for anaplastic small cell carcinoma of the bronchus: a randomised, controlled trial.. Cancer Chemother Pharmacol.

[OCR_01289] Fayers P. M., Jones D. R. (1983). Measuring and analysing quality of life in cancer clinical trials: a review.. Stat Med.

[OCR_01294] Feld R., Evans W. K., DeBoer G., Quirt I. C., Shepherd F. A., Yeoh J. L., Pringle J. F., Payne D. G., Herman J. G., Chamberlain D. (1984). Combined modality induction therapy without maintenance chemotherapy for small cell carcinoma of the lung.. J Clin Oncol.

[OCR_01299] Feld R., Pringle J. F., Evans W. K., Keen C. W., Quirt I. C., Curtis J. E., Baker M. A., Yeoh J. L., Deboer G., Brown T. C. (1981). Combined modality treatment of small cell carcinoma of the lung.. Arch Intern Med.

[OCR_01304] Greco F. A., Johnson D. H., Hainsworth J. D., Wolff S. N. (1985). Chemotherapy of small-cell lung cancer.. Semin Oncol.

[OCR_01333] Simon R. (1982). Patient subsets and variation in therapeutic efficacy.. Br J Clin Pharmacol.

